# The Greater the Tilt, the Taller the Nest? The Effect of Solar Array Type on Bird Nest Architecture and Nest Microclimate

**DOI:** 10.1002/ece3.71539

**Published:** 2025-06-16

**Authors:** Brendan Enochs, Adrianna Burghardt, Amy Friemoth, Chelse Prather

**Affiliations:** ^1^ Department of Biological Sciences University of Dayton Dayton Ohio USA

**Keywords:** grassland, nest building, photovoltaic, solar array, solar prairie

## Abstract

The increasing demand for renewable energy has led to widespread installation of photovoltaic (PV) solar arrays, however, their ecological impacts, particularly on bird species, remain poorly understood. This study investigates robin nesting behavior within fixed and tracking solar arrays in Dayton, Ohio, with a focus on species presence, nest architecture modifications, and thermal conditions. We found American robin ( *Turdus migratorius* ), House finch ( *Haemorhous mexicanus* ), and House sparrow ( *Passer domesticus* ) nests across two sites: a tracking array and a fixed array. Results showed that robin nests in the tracking array exhibited significant architectural adaptations, including taller nests and greater variability in attachment angles, likely to enhance stability as panels moved throughout the day. Additionally, nests in tracking arrays experienced warmer thermal conditions than those in fixed arrays. These findings suggest that generalist bird species can adapt to nesting in dynamic, human‐modified environments, modifying their nests to cope with structural movement and thermal changes. The study contributes to understanding bird ecology in solar arrays and provides insights for integrating wildlife considerations into solar energy designs.

## Graphical abstract

Solar energy is increasing at an exponential rate, but little is known about how birds respond to solar arrays, including their nest‐building behaviors. Solar arrays can be tilting that move to face the sun or fixed arrays that do not move. We identified and measured the dimensions of nests built on solar array infrastructure in both a tilting array and a fixed array. We observed only generalist bird nests on the solar arrays, and nests on tilting arrays were taller and hotter than on fixed arrays. Our results suggest that generalist species use solar arrays and that nest architecture is altered due to solar array type. Future studies should determine how arrays affect hatching and fledgling success on photovoltaic arrays.

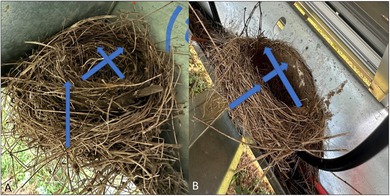

## 1 Introduction

In recent years, the increasing efficiency and decreasing costs of photovoltaic (PV) solar panels, combined with the urgent need for renewable energy to combat global climate change, have led to an exponential rise in solar array installations (Ghosh and Yadav 2021; International Energy Agency 2023). PV cells convert sunlight into electrical current and are used on a large scale in solar arrays that consist of multiple interconnected rows of panels. These panels can be composed of various types of cells and may either remain fixed at one angle throughout the day or be designed to tilt to different angles (fixed arrays), tracking the sun's movement for optimal solar energy absorption (tracking arrays). While these tracking arrays adjust dynamically to capture maximum sunlight, fixed arrays remain stationary, receiving solar radiation at less direct angles.

The construction of these large‐scale projects often involves clearing vegetation from natural or converted habitats (Hernandez et al. 2014), yet the full extent of their ecological impact on wildlife is still not well understood (Hartmann et al. 2016). Additionally, there is growing interest in transforming the land beneath solar arrays into more biodiverse habitats (Hernandez et al. 2019), a concept known as “ecovoltaics” (Sturchio and Knapp 2023), and the resulting habitats are often called solar prairies. However, little guidance exists on how to effectively create such habitats to attract wildlife.

Some studies have shown that solar arrays can support a variety of wildlife (Graham et al. 2021; Nordberg et al. 2021; Lafitte et al. 2023), including birds, which can be affected in multiple ways. Some studies have noted bird mortality associated with the construction of PV arrays (Kagan et al. 2014; Visser et al. 2019). Research by DeVault et al. (2014) showed that, while bird species diversity was lower in and around PV arrays compared to adjacent grasslands, bird density within solar arrays was nearly double that of nearby grasslands. This suggests that generalist species—those adapted to diverse habitats, food resources, and environmental conditions—may thrive in solar arrays, a trend seen more broadly across human‐modified landscapes (Devictor et al. 2007).

Nest architecture—including geometry, attachment angles, and height—varies widely among bird species and is influenced by environmental conditions, predator pressure, and the need for structural stability (Hansell 2000). Birds construct nests suited to their habitats, with variation in shape and attachment reflecting selective pressures such as wind exposure, substrate type, and available materials (Hansell 2000). In dynamic or exposed environments, birds often build more robust, deeper, or elevated nests to maintain structural integrity and ensure reproductive success (Hansell 2000; Deeming and Mainwaring 2015). Additionally, nest orientation and attachment strategies are adapted to reduce mechanical stress from wind or movement in the nesting substrate—adaptations that become especially relevant in human‐altered environments, where natural supports are replaced by artificial ones (Hansell 2000; Deeming and Mainwaring 2015).

Interestingly, some bird species have been observed nesting on the structures supporting solar panels (Hernandez et al. 2014) or on the panels themselves, as noted in this study, across both fixed and tracking arrays. Nesting behaviors, essential for population persistence, often change in response to human‐dominated environments. Birds are known to adjust the timing of reproduction and incorporate anthropogenic materials into their nests in novel habitats (Bressler et al. 2020), yet it remains unclear whether they also modify the physical architecture of their nests to adapt to new settings (Reynolds et al. 2019). The unique movement of tracking solar arrays offers an opportunity to investigate whether birds alter their nest architecture to suit such dynamic environments. Birds nesting in tracking arrays may need to modify their building techniques and attachment strategies to maintain nest stability as the panels move throughout the day.

Additionally, the differences in solar energy intake between tracking and fixed arrays may create distinct thermal environments for nests. Thermal conditions are critical to nesting success, as even small deviations from optimal temperatures can influence incubation efficiency, hatching success (Mainwaring 2015), and chick development (Conway and Martin 2000; Perez et al. 2020). Temperature variation within and among nest sites has been shown to affect parental behavior and reproductive outcomes, underscoring the importance of nest microclimate in shaping fitness‐related traits (Ardia 2006). Temperature differences created by solar infrastructure have the potential to affect survival and growth from embryo to hatchling. While it is known that the air temperature underneath the panels is cooler than the surrounding environment (Armstrong et al. 2016; Lambert et al. 2021; Graham et al. 2021), it is not known how nest temperature might be altered directly under the panels where the birds are nesting so close to panels that get hotter than ambient temperatures during the day. It is also unclear how nest temperature may differ between fixed and tracking arrays, or how this temperature difference could alter nest architecture. Understanding the relationship between nest architecture and thermal conditions can provide valuable insights into how birds adapt to nesting in solar arrays.

This study focuses on three key questions:

1. Which bird species are utilizing solar arrays for nesting?
2. Do birds modify nest architecture in response to the movement of tracking panels?
3. How does the type of solar array affect the thermal conditions within nests?

We hypothesized that nests in tracking arrays would display architectural adaptations—such as greater height from the top of the nest to the bottom and more varied slopes (slope of the top of the nest relative to the ground) of the nest—to maintain egg stability during movement. Specifically, we predicted that taller nests in tracking arrays would help prevent eggs from falling when panels tilt steeply, and that more varied nest slopes would occur from the panel movement throughout the day during construction. Lastly, we expected tracking arrays to create warmer thermal environments for nests due to their ability to track the sun and absorb maximum solar radiation. Note that we did not compare these solar sites to reference sites outside of solar arrays; the purpose of this study was just to look at nest architecture and temperature between a fixed and a tracking array, but we would expect these parameters to differ from nests on natural materials.

## 2 Methods

Two peri‐urban sites in Dayton, Ohio were selected for this study: Curran Place (39.73° N, 84.19° W), which has a monofacial tracking solar array built in 2018, and the Marianist Environmental Education Center (MEEC; 39.72° N, 84.10° W), which hosts a monofacial fixed solar array constructed in 2023. The arrays were 8.17 km apart, and Curran Place is slightly larger (4.5 ha) than MEEC (3 ha).

After visiting each site in late August 2023, all intact abandoned bird nests were marked with red flagging tape. Since bird nests were typically removed from solar arrays after the nesting season to reduce fire risks, these nests had been constructed during the previous spring, and whether they were successfully used for breeding was unknown. Nests were not active when measured, but since they had been constructed at the same time, it was assumed that any degradation had occurred at the same rate. Curran Place was revisited on September 25, 2023, and MEEC in the following week, and the architecture of each flagged nest was measured using calipers (Carbon Fiber Composites). Nest height was recorded from the base of the nest to the top, and two diameters were measured (front‐to‐back and side‐to‐side) to capture any variability in the shape of each nest. The nest slope was measured using the iPhone level app. The nest slope was taken by placing an iPhone against the top of the nest. A measurement of 0° indicated that the top of the nest was parallel to the ground, while a measurement greater than 0° indicated the top of the nest was sloped with respect to the ground. Photos of nests were taken to assist in species identification.

To assess the thermal conditions of nests, iButtons were placed at the bottom of the nest cavity in five randomly selected American robin nests at each site on September 25, 2023. These iButtons recorded temperature every 30 min for 15 days. Only American robin ( *Turdus migratorius* ) nests were used for thermal data collection because this was the only species present at both sites. Since iButtons were deployed in the fall, the temperatures recorded do not directly reflect thermal conditions experienced by nests during the breeding season but rather serve as a preliminary estimate of the potential temperature differences between the two solar array types.

All data analysis was conducted in R version 4.2.2 (R Core Development Team, 2023). Welch's ANOVAs were performed to compare the dimensions of the most common species, the American robin, nest height, slope, diameter (side‐to‐side), and diameter (front‐to‐back) between the two panel types. Welch's ANOVAs were chosen because they do not assume equal variances between groups (Welch 1951). To assess potential differences in the thermal properties of nests in fixed and tracking solar arrays, the daily maximum and mean temperature for each iButton was calculated, and separate Gaussian generalized linear mixed effects models (GLMMs) using the R package *lme4* (version 1.1‐32; Bates et al. 2015) were employed. In these two models, the response variable was daily maximum or mean temperature and included a fixed effect for array panel type (fixed or tracking). Random intercepts for date and ibutton were also included in both models.

## 3 Results

**FIGURE 1:**
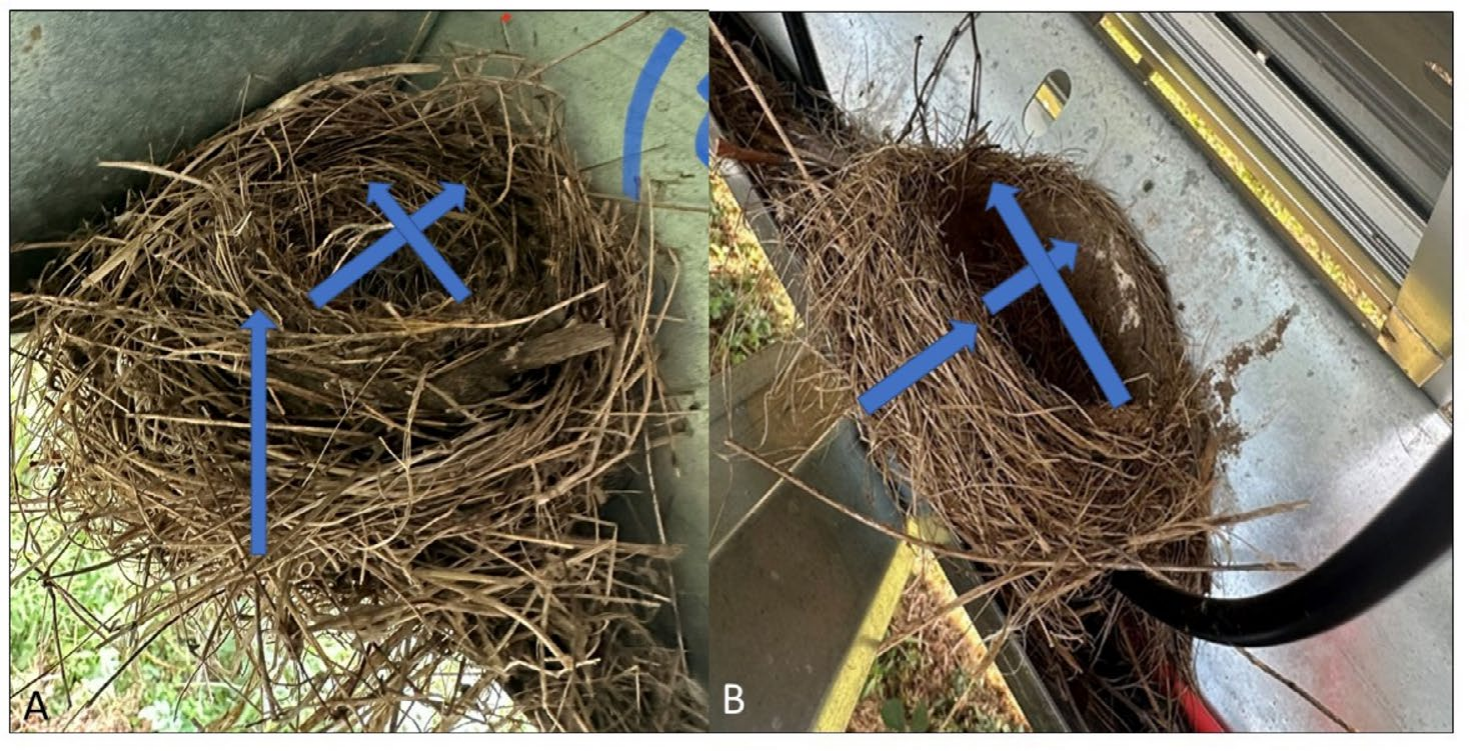
*Turdus migratorius* nest on a tracking array (A) versus a fixed array (B). Nests on tilting arrays are taller and have a higher angle of attachment than nests on fixed arrays. Arrows indicate diameter and height measurements.

At the tracking solar array at Curran Place, we observed 49 nests (10.9 nests/ha) from three generalist bird species: American robin ( *Turdus migratorius* ; *n* = 9), House finch ( *Haemorhous mexicanus* ; *n* = 22), and House sparrow ( *Passer domesticus* ; *n* = 18). At the fixed solar array at MEEC, we recorded only *T. migratorius* nests (*n* = 18; 6 nests/ha). All nests were built on horizontal metal beams directly underneath the panels. At the tracking array, all robin nests were built at the corners where two supporting beams came together. In contrast, at the fixed array, robin nests were built in the middle of beams, often alongside cables (see Figure 1 for pictures of nests in both systems).

**FIGURE 2:**
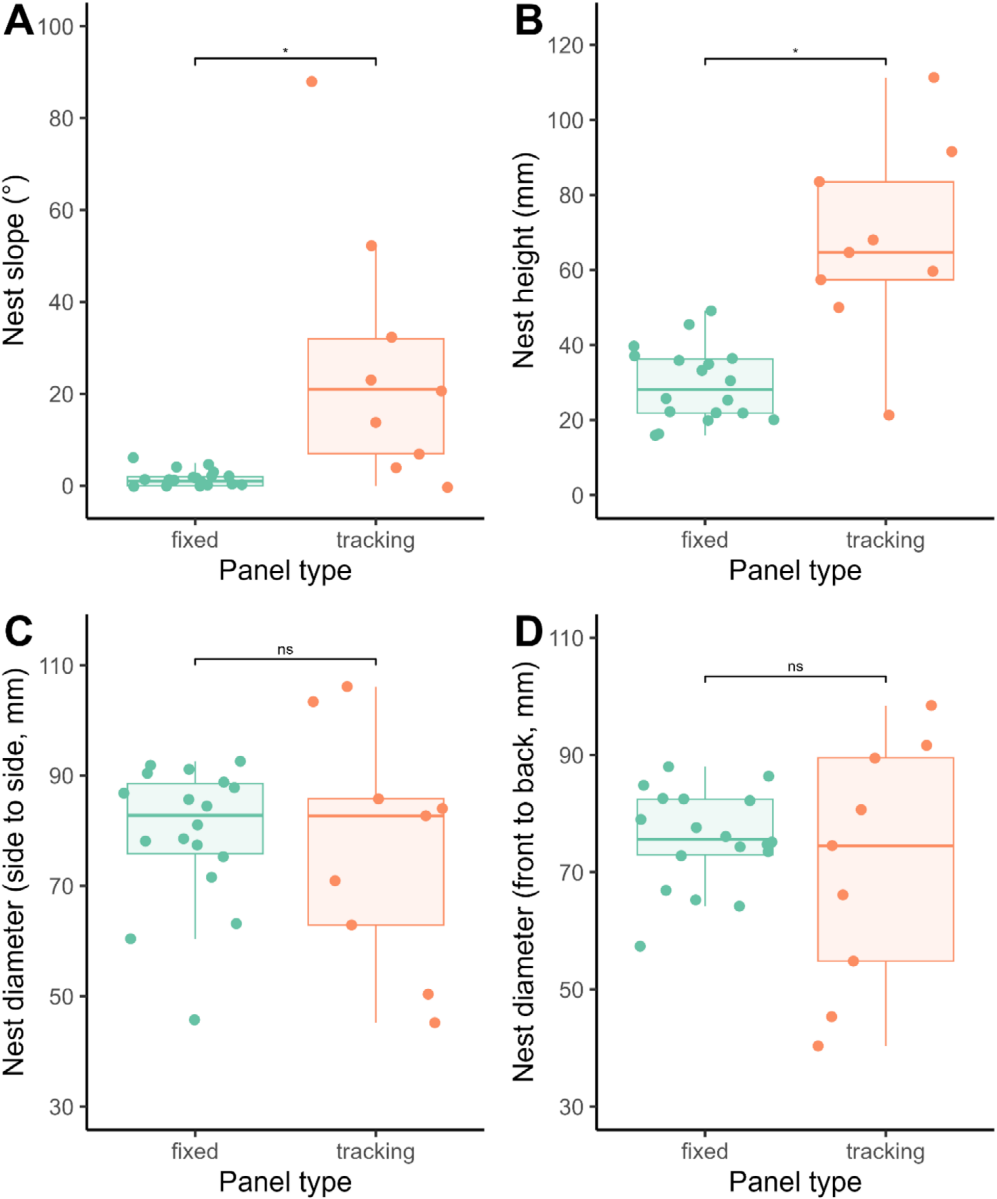
Comparisons of robin nest architecture between tracking and fixed solar arrays with regard to (A) nest slope, (B) nest height, (C) nest diameter (front‐to‐back), and (D) nest diameter (side‐to‐side).

The American robin was the only species found to nest at both the fixed and tracking array sites, so all comparisons in nest architecture between array types were conducted using this species alone. The mean slope of robin nests differed between tracking and fixed panels (*F*_1,8.03_ = 7.25, *p* = 0.027), with tracking array nests exhibiting greater variability and a mean slope of 26.8° (SD = 27.9), compared to a mean of 1.7° (SD = 1.81) for nests at the fixed array (Figure 2A). The mean height of robin nests also differed between the tracking array (mean = 67.5 mm, SD = 25.9) and fixed array (mean = 29.5 mm, SD = 9.93) (*F*_1,9.20_ = 18, *p* = 0.002; Figure 2B). On the other hand, no differences in robin nest diameter were observed between the two array types in either orientation (side‐to‐side: *F*_1,10.9_ = 0.12, *p* = 0.74; front‐to‐back: *F*_1,9.27_ = 0.38, *p* = 0.55; Figure 2C,D). The side‐to‐side nest diameter averaged 79.5 mm (SD = 12.6) on fixed arrays and 76.8 mm (SD = 21.4) on tracking arrays, whereas the front‐to‐back diameter averaged 75.7 mm (SD = 8.29) on fixed arrays and 71.2 mm (SD = 21) on tracking arrays.

**FIGURE 3:**
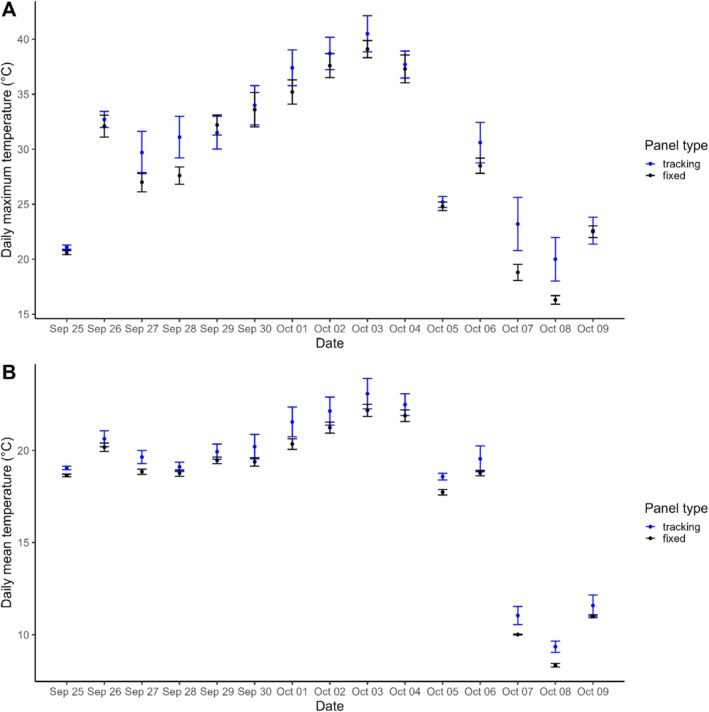
Daily mean temperature (A) and daily maximum temperature (B) of nests at tracking and fixed solar arrays across 15 days. Each point is the average of five iButtons placed in nests at each array type, and the vertical bars show the corresponding 95% confidence intervals.

Temperature data revealed considerable variation in nest microclimates over the 15‐day period. Across both sites, daily mean temperatures ranged from 8.2°C to 24.2°C, while daily maximum temperatures ranged from 16.0°C to 43.0°C. The daily mean temperature within nests varied between the fixed and tracking array (*β* = −0.74, SE = 0.26, *p* = 0.020), with nests at the tracking array being, on average, 0.74°C warmer than nests at the fixed array (Figure 3A). In contrast, the daily maximum temperature within nests did not differ between the fixed and tracking array (*β* = −1.52, SE = 0.76, *p* = 0.081; Figure 3B). For both daily mean and maximum temperature models, the random effect for day accounted for substantial temperature variation (SD = 4.33°C and 6.91°C, respectively).

## 4 Discussion

The nesting ecology of birds in human‐dominated landscapes is a relatively unexplored area, particularly in relation to solar arrays (Reynolds et al. 2019). Our findings support the idea that generalist bird species, such as American robin, House finch, and House sparrow, utilize solar arrays for nesting. These results are consistent with the broader trend that generalists tend to thrive in novel environments (Devictor et al. 2007; Kurucz et al. 2021). The difference in the number of generalist species could be due to the difference in age of the two arrays—the tracking array had only had 1 year for birds to colonize, which is likely why we only found 1 species, American robins, at this site.

Birds that inhabit and nest in urban landscapes often exhibit adjusted behaviors (Bressler et al. 2020); however, few empirical studies have identified alterations in nest architecture beyond changes in the building materials used (Reynolds et al. 2019). As hypothesized, American robins nesting in tracking solar arrays displayed architectural modifications, constructing taller and more inclined nests. These changes likely serve to improve nest stability in response to the dynamic movement of tracking panels. Specifically, taller nests may help prevent eggs from rolling out, while altering the slope of the nest relative to the ground may help counteract the panel's periodic tilt, reducing the risk of displacement during movement. In terms of nest size, our measurements revealed that robin nests in solar arrays (mean nest height: fixed = 28.1 mm and tracking = 64.1 mm) were smaller than those typically reported in natural habitats, which range between 75 and 106 mm (Iowa, Pennsylvania; Howell 1942) but is highly variable across the species' range (40–270 mm; McFarland et al. 2021). To our knowledge, this is the first documented instance of such adaptive nesting behaviors associated with solar arrays.

Nest temperature strongly influences chick growth and survival, with smaller nests considered more adaptive in warmer environments (Perez et al. 2020). The potentially warmer microclimate beneath solar panels may reduce the need for larger, more insulated nests, resulting in the smaller robin nests observed within both arrays. The lack of measurements of reference temperatures from nests on natural material at both sites limited our ability to better understand the relationship between nest size and temperature, but generally, they were fairly close, had similar land use cover surrounding them, and experienced similar weather. However, the greater height of nests in tracking arrays suggests that nest architecture is also influenced by factors beyond temperature, such as the need to prevent eggs from falling during panel movement. As we did not measure nests in reference areas outside of the solar arrays, we could not compare nest architecture in nests on natural materials to solar arrays; however, this could further elucidate the differences we observed.

Further research is needed to explore how other factors, such as predation risk, may influence fledgling success in solar arrays. Although the species we found can nest under solar arrays, this does not mean that they are successfully breeding there. Comparing the success of nests in solar arrays with those in nearby natural habitats, while incorporating predation risk assessments, would help disentangle the drivers of how nest size variation, thermal environments, and fitness outcomes are related in these novel environments. For instance, could higher temperatures underneath panels undermine hatching success? Additionally, one may predict that while higher nests in tracking arrays may ameliorate the detrimental effects of nest movement throughout the day on nest success, their proximity to the ground and over vegetation may increase predation risk when nesting on any type of solar array. By documenting the unique nesting conditions and changes to nests in tracking arrays, we provide insights that could inform the design of ecovoltaic systems that better accommodate wildlife, especially once further studies clarify how these changes relate to fledgling success.

## Author Contributions

**Brendan Enochs:** data curation (lead), formal analysis (lead), investigation (lead), methodology (lead). **Adrianna Burghardt:** formal analysis (supporting), investigation (supporting), methodology (supporting), writing – review and editing (supporting). **Amy Friemoth:** data curation (supporting), formal analysis (supporting), investigation (supporting), methodology (supporting), visualization (supporting), writing – review and editing (supporting). **Chelse Prather:** conceptualization (lead), methodology (equal), resources (lead), writing – review and editing (lead).

## Conflicts of Interest

The authors declare no conflicts of interest.

## Data Availability

Data and code can be found on Figshare: https://doi.org/10.6084/m9.figshare.27855363.v1.
